# Osteopathic Manipulative Treatment Protocol for Postoperative Care Following Abdominal Surgery: A Quality Improvement Project

**DOI:** 10.7759/cureus.98685

**Published:** 2025-12-08

**Authors:** Joanne Genewick, Alfred Amendolara, Sara Robinson, Michelle McDonough, Marisela Loera, Stephen K Stacey

**Affiliations:** 1 Family Medicine, Mayo Clinic Health System, Mankato, USA; 2 Neurology, St. Luke's University Health Network, Bethlehem, USA; 3 Biomedical Sciences, Noorda College of Osteopathic Medicine, Provo, USA; 4 Family Medicine, Mayo Clinic Health System, Mankota, USA; 5 Family Medicine, Mayo Clinic Health System, La Crosse, USA

**Keywords:** general surgery, omt, osteopathic manipulative medicine, post-operative ileus, quality improvement

## Abstract

Background

Postoperative ileus (POI) is a common condition that increases length of stay (LOS) and resource utilization. However, treatment consists primarily of supportive care. No workflow addressing measures to promote early bowel function recovery following abdominal surgery was in place at Mayo Clinic Health System Hospital in Mankato. Osteopathic manipulative treatment (OMT) may provide a cost-effective and low-risk option for reducing LOS of surgical patients. The primary aim of this quality improvement project was to decrease LOS of patients at risk for POI following abdominal surgery by implementing a standardized OMT protocol at Mayo Clinic Health System Hospital in Mankato, MN.

Methods

This was a single-center, non-randomized, mixed retrospective-prospective quality improvement project taking place from 2021-2023 taking place at Mayo Clinic Health System Hospital in Mankato, MN. Surgical patients admitted for abdominal surgery with an expected length of stay greater than a day were invited to participate and receive treatment with a pre-defined OMT treatment protocol including seven techniques. Patients under 18 were excluded. A total of 33 patients were enrolled over two cycles, February 1, 2022 - April 30, 2022, and February 1, 2023 - April 30, 2023. A baseline comparison group of 55 patients was retrospectively compiled following these same criteria over a period from January 1, 2021, to April 30, 2021. The primary outcome measured was LOS in days. Secondary outcomes were time to first bowel movement, time to first flatus, 30-day readmission rate, and postoperative LOS.

Results

The baseline group had a median LOS of 7.0 days and while the OMT group had a median LOS of 6.0 days resulting in a non-significant median difference of -1 days [Hodges-Lehmann estimator: -1, 95% CI (-3.0, 0.00), *p*=0.128] as well as an effect size *r*=-0.19 [95% CI (-0.44, 0.03)] favoring the OMT group. No significant difference was found in secondary outcomes. Subgroup analysis revealed a significant reduction in median LOS [Hodges-Lehmann estimator: -2.99, 95% CI (-5.00, -1.00), p=0.011, r=-0.38] and postoperative LOS [Hodges-Lehmann estimator: -2.00, 95% CI (-3.99, 0.00), *p*=0.028, r=-0.33] in patients who underwent open surgical procedures.

Conclusions

In this quality improvement (QI) initiative, the incorporation of a standardized OMT protocol was associated with a non-significant reduction in LOS and postoperative LOS. However, patients undergoing open surgical procedures did show a significant reduction in both LOS and postoperative LOS. These findings suggest the feasibility of this protocol; however, due to the limitations of the study, they warrant further investigation in properly powered, randomized-controlled trials.

## Introduction

Postoperative ileus (POI)-the temporary cessation of intestinal motility in the absence of obstruction-is a common complication following abdominal surgery. It prolongs hospital stays and substantially drives up healthcare costs, yet no standardized intervention exists to reliably reduce its impact. Recognizing this gap, we designed a quality improvement (QI) protocol involving the use of osteopathic manipulative treatment (OMT) aimed at reducing length of stay (LOS) in patients at high risk of POI.

Postoperative ileus affects 10-30% of postoperative patients and can lead to nausea, vomiting, distension, and bloating. While there is no clear consensus, the duration is typically considered pathological if it persists longer than 72 hours. The pathophysiology of POI is multifactorial, involving sympathetic nervous system activation, inflammation, surgical stress, and opioid-related gut dysmotility. Identified risk factors include age, male sex, increased blood loss, and surgical techniques [1-4].

Postoperative ileus is a leading cause of prolonged hospital stays following abdominal surgery, which is costly to hospitals and patients [5]. In a retrospective study of 17,876 colectomy patients, those with POI experienced a mean length of stay in the hospital of 13.8 days compared with 9.5 days in those without POI [6]. The associated cost increase in healthcare expenditures has been estimated to exceed $10,000 per episode based on data from 2016 [7], with the total cost of POI treatment across the United States lying between $7.5 million to $1 billion annually [8]. While protocols such as enhanced recovery after surgery (ERAS) have shown consistent benefit in length of stay and morbidity following surgical interventions, including reduction in time to global resumption of intestinal function, direct treatments for POI remain limited. Most cases are managed with supportive care or prokinetic agents [3,9-12]. Given the large burden of POI, additional treatment options may provide substantial monetary and resource savings.

One promising treatment for POI is OMT [13,14], which is “the therapeutic application of manually guided forces by an osteopathic physician to improve physiologic function and/or support homeostasis that has been altered by somatic dysfunction” [15]. Numerous OMT techniques are hypothesized to modulate sympathetic tone, enhance parasympathetic activity, and improve lymphatic return. Based on current understanding of POI pathophysiology, OMT techniques targeting the nervous system, lymphatic system, or gut might be hypothesized to improve recovery and prevent POI. Preclinical and clinical studies suggest that such techniques result in earlier bowel movements in animal models [16], reduce hospital length of stay (11.3 days in the OMT group vs. 17.4 days in the non-OMT group) [17], and reduce postoperative pain following abdominal surgeries [18]. OMT therefore provides an attractive, low-cost, and low-risk intervention for treating POI [19-21]. However, definitive evidence of clinical effectiveness remains lacking.

No standardized workflow was in place to specifically address POI in postoperative care. Previous research had shown preliminary success of a protocol incorporating OMT into the treatment and prevention of POI. Thus, given the clinical and economic burden of POI and the availability of proficient osteopathic providers at the hospital, we implemented a protocol incorporating OMT for patients undergoing abdominal surgery. The primary aim of this quality improvement project was to decrease hospital length of stay among patients at risk for POI following abdominal surgery by implementing an OMT protocol at Mayo Clinic Health System in Mankato.

## Materials and methods

This quality improvement project was completed at Mayo Clinic Health System Hospital in Mankato. It was approved by the Mayo Clinic Quality Academy (Project ID 3997) and has been reported following the Standards for Quality Improvement Reporting and Excellence (SQUIRE) 2.0 guidelines [22]. Data was collected from February 1, 2022, to April 30, 2023. No conflicts of interest were identified before, during, or after the project period. The Epic Systems electronic health record (EHR) (Epic Systems Corporation, Verona, USA) was used to access all patient records and retrospective data. Prospective patient data was collected via a daily survey of the general surgery census at Mayo Clinic Health System Hospital in Mankato and was manually compiled once patients were identified and enrolled. Baseline data were collected via an Epic report based on a predetermined list of procedure codes (Table 1). All patients admitted during the baseline period with the specified International Classification of Diseases (ICD) codes were included in the comparison group. 

**Table 1:** List of included ICD diagnoses ICD: International Classification of Diseases.

ICD Diagnosis
SIGMOID COLECTOMY WITH STOMA
SIGMOID COLECTOMY WITH ANASTOMOSIS
LAPAROSCOPIC SIGMOID COLECTOMY WITH STOMA
OPEN SIGMOID COLECTOMY LOW ANTERIOR RESECTION
ROBOTIC-ASSISTED SIGMOID COLECTOMY WITH STOMA
LAPAROSCOPIC SIGMOID COLECTOMY WITH ANASTOMOSIS
ROBOTIC-ASSISTED SIGMOID COLECTOMY WITH ANASTOMOSIS
LAPAROSCOPIC SIGMOID COLECTOMY LOW ANTERIOR RESECTION
HAND-ASSISTED LAPAROSCOPIC SIGMOID COLECTOMY WITH STOMA
ROBOTIC-ASSISTED SIGMOID COLECTOMY LOW ANTERIOR RESECTION
HAND-ASSISTED LAPAROSCOPIC SIGMOID COLECTOMY WITH ANASTOMOSIS
DIAGNOSTIC LAPAROSCOPY
ROBOTIC-ASSISTED LYSIS OF ADHESIONS
LAPAROSCOPIC ABDOMINAL EXPLORATION - LYSIS OF ADHESIONS
OPERATIVE LAPAROSCOPY-LYSIS OF ADHESIONS
LYSIS ADHESIONS OVARY, EXPLORATION ABDOMINAL - LYSIS ADHESIONS
EXPLORATORY LAPAROTOMY
LAPAROTOMY - EXPLORATORY, OPERATIVE LAPAROSCOPY
COLECTOMY LEFT WITH STOMA
COLECTOMY RIGHT WITH STOMA
COLECTOMY WITH STOMA – TOTAL
COLECTOMY LEFT WITH ANASTOMOSIS
COLECTOMY TRANSVERSE WITH STOMA
COLECTOMY RIGHT WITH ANASTOMOSIS
COLECTOMY WITH ANASTOMOSIS – TOTAL
COLECTOMY TRANSVERSE WITH ANASTOMOSIS
SUBTOTAL COLECTOMY WITH STOMA
EXTENDED COLECTOMY LEFT WITH STOMA
EXTENDED COLECTOMY RIGHT WITH STOMA
SUBTOTAL COLECTOMY WITH ANASTOMOSIS
LAPAROSCOPIC COLECTOMY LEFT WITH STOMA
LAPAROSCOPIC COLECTOMY RIGHT WITH STOMA
LAPAROSCOPIC TOTAL COLECTOMY WITH STOMA
EXTENDED COLECTOMY LEFT WITH ANASTOMOSIS
EXTENDED COLECTOMY RIGHT WITH ANASTOMOSIS
LAPAROSCOPIC SUBTOTAL COLECTOMY WITH STOMA
ROBOTIC-ASSISTED COLECTOMY LEFT WITH STOMA
ROBOTIC-ASSISTED COLECTOMY RIGHT WITH STOMA
ROBOTIC-ASSISTED TOTAL COLECTOMY WITH STOMA
LAPAROSCOPIC COLECTOMY LEFT WITH ANASTOMOSIS
LAPAROSCOPIC COLECTOMY RIGHT WITH ANASTOMOSIS
LAPAROSCOPIC TOTAL COLECTOMY WITH ANASTOMOSIS
LAPAROSCOPIC EXTENDED COLECTOMY LEFT WITH STOMA
LAPAROSCOPIC COLOSTOMY CLOSURE
ROBOTIC-ASSISTED COLOSTOMY CLOSURE
HAND-ASSISTED LAPAROSCOPIC COLOSTOMY CLOSURE
CLOSURE COLOSTOMY
RESECTION SMALL INTESTINE WITH ANASTOMOSIS
ROBOTIC-ASSISTED SMALL BOWEL RESECTION
LAPAROSCOPIC SMALL BOWEL RESECTION WITH STOMA
ROBOTIC-ASSISTED SMALL BOWEL RESECTION WITH STOMA
RESECTION SMALL BOWEL WITH STOMA
LAPAROSCOPIC EXPLORATION – DIAGNOSTIC
LAPAROSCOPIC ABDOMINAL EXPLORATION
LAPAROSCOPIC BILE DUCT EXPLORATION WITH CHOLEDOCHODUODENOSTOMY
LAPAROSCOPIC ABDOMINAL EXPLORATION - EVACUATION OF HEMATOMA - CONTROL HEMORRHAGE
ROBOTIC-ASSISTED TOTAL COLECTOMY WITH ANASTOMOSIS
ROBOTIC-ASSISTED COLECTOMY RIGHT WITH ANASTOMOSIS
ROBOTIC-ASSISTED TRANSVERSE COLECTOMY WITH STOMA
LAPAROSCOPIC TRANSVERSE COLECTOMY WITH ANASTOMOSIS
HAND-ASSISTED LAPAROSCOPIC COLECTOMY LEFT WITH STOMA
HAND-ASSISTED LAPAROSCOPIC COLECTOMY RIGHT WITH STOMA
HAND-ASSISTED LAPAROSCOPIC TOTAL COLECTOMY WITH STOMA
LAPAROSCOPIC EXTENDED COLECTOMY LEFT WITH ANASTOMOSIS
ROBOTIC-ASSISTED EXTENDED COLECTOMY LEFT WITH STOMA
ROBOTIC-ASSISTED EXTENDED COLECTOMY RIGHT WITH STOMA
ROBOTIC-ASSISTED PARTAIL COLECTOMY WITH ANASTOMOSIS
ROBOTIC-ASSISTED SUBTOTAL COLECTOMY WITH ANASTOMOSIS
LAPAROSCOPIC EXTENDED COLECTOMY RIGHT WITH ANASTOMOSIS
ROBOTIC-ASSISTED TRANSVERSE COLECTOMY WITH ANASTOMOSIS
HAND-ASSISTED LAPAROSCOPIC SUBTOTAL COLECTOMY WITH STOMA
ROBOTIC-ASSISTED EXTENDED COLECTOMY LEFT WITH ANASTOMOSIS
HAND-ASSISTED LAPAROSCOPIC COLECTOMY LEFT WITH ANASTOMOSIS
HAND-ASSISTED LAPAROSCOPIC COLECTOMY RIGHT WITH ANASTOMOSIS
HAND-ASSISTED LAPAROSCOPIC TOTAL COLECTOMY WITH ANASTOMOSIS
ROBOTIC-ASSISTED EXTENDED COLECTOMY RIGHT WITH ANASTOMOSIS
HAND-ASSISTED LAPAROSCOPIC SUBTOTAL COLECTOMY WITH ANASTOMOSIS
HAND-ASSISTED LAPAROSCOPIC TRANSVERSE COLECTOMY WITH ANASTOMOSIS
HAND-ASSISTED LAPAROSCOPIC EXTENDED COLECTOMY LEFT WITH ANASTOMOSIS
HAND-ASSISTED LAPAROSCOPIC EXTENDED COLECTOMY RIGHT WITH ANASTOMOSIS
ROBOTIC-ASSISTED COLECTOMY LEFT WITH ANASTOMOSIS
LAPAROSCOPIC SUBTOTAL COLECTOMY WITH ANASTOMOSIS
LAPAROSCOPIC EXTENDED COLECTOMY RIGHT WITH STOMA
ROBOTIC-ASSISTED SUBTOTAL COLECTOMY WITH STOMA
CLOSURE ILEOSTOMY
REVISION ILEOSTOMY
OPEN SIGMOID LOW ANTERIOR RESECTION WITH STOMA

Setting

Mayo Clinic Health System Hospital in Mankato is a 118-bed Level III trauma center in Mankato, Minnesota, and is a regional hospital within a large multi-specialty health system in the Midwest. The hospital is home to the Mayo Clinic Family Medicine Residency-Mankato program, which has a total of 15 residents. This residency program maintains osteopathic recognition and includes osteopathic education and training for all residents enrolled in the osteopathic recognition curriculum. No previous attempts to reduce post-operative length of stay using osteopathic manipulation have been made. This QI project was supported by hospital administration, residency administration, and the surgical department.

Participants

**Figure 1:**
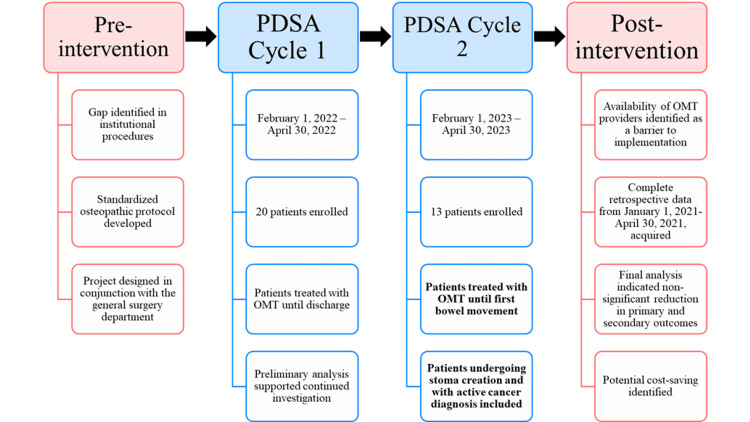
Plan-do-study-act flowchart This flowchart details the project timeline, including pre-planning, two plan-do-study-act (PDSA) cycles, and the post-intervention analysis. Changes between cycles are shown in bold text.

Patients were enrolled during two prospective QI cycles structured using the plan-do-study-act (PDSA) framework: February 1, 2022, to April 30, 2022 (Cycle 1) and February 1, 2023, to April 30, 2023 (Cycle 2) (Figure 1). All eligible patients admitted during each cycle were invited to participate via consecutive sampling. Patients who agreed to treatment were enrolled. Patients declining treatment were not included in the analysis. Verbal consent for participation was obtained from all prospective patients and documented in the EHR. Demographic data were obtained via an EHR query. Eligibility criteria included: (1) receipt of surgery for any of the prespecified diagnoses (Table 1), (2) age over 18, (3) expected length of stay greater than a day, (4) no active cancer, and (5) not undergoing cholecystectomy, appendectomy, or stoma creation. Criteria were developed in collaboration with general surgeons at the study site. Based on stakeholder feedback during Cycle 1, inclusion criteria were broadened for Cycle 2 to include patients who were undergoing stoma creation and patients with active cancer diagnoses. No other modifications to the recruitment strategy or eligibility criteria were made.

Intervention

A standardized OMT treatment plan was developed to address the hypothesized physiological processes underlying POI by normalizing sympathetic stimulation and improving lymphatic flow and drainage to reduce inflammation [4,23]. The treatment protocol comprised seven techniques: muscle energy for the occipitoatlantal joint, myofascial release of the thoracic inlet, paraspinal myofascial release, abdominal diaphragm myofascial release, pelvic diaphragm myofascial release, sacral rocking, and pedal lymphatic pump. Treatment sessions lasted approximately 20-30 minutes. Every treatment was performed once in sequence during each treatment session. Descriptions of each treatment may be found in Appendices 1, 2, 3. In addition to the OMT protocol described here, all patients in the intervention and baseline groups received standard pre-, intra-, and post-op care following ERAS recommendations. No attempt at blinding of the intervention was made. Treatment adherence was reviewed based on in-chart documentation of OMT treatment. 

Treatments were delivered by residents at the Mayo Clinic Family Medicine Residency-Mankato program (MM, SR, ML, LD, TP, AV, KS, KL). Participating residents were designated osteopathic residents who had previously demonstrated competency in OMT. To ensure standardized application, treating providers received at least 45 minutes of training in the protocol techniques before the start of the project. 

During Cycle 1, OMT was delivered once daily from postoperative day one until discharge. During Cycle 2, the protocol was modified such that patients received OMT once daily from postoperative day one until the day of their first bowel movement (Figure 1). This change was implemented to reduce the time and cost of the intervention. Patients in both the intervention and baseline groups otherwise received usual pre- and post-operative care.

Study of the intervention

In order to determine the impact of the implemented protocol, a prospective, non-randomized design including a retrospective historical control group was implemented. The retrospective cohort of patients was compiled by screening all patient records with included diagnosis codes (Table 1) in the EHR from a period one year earlier than the initial PDSA cycle (January 1, 2021, to April 30, 2021). All patients matching the inclusion criteria detailed above and admitted during that period were included in the comparison group. 

Measures and outcomes

The primary outcome was the total length of stay (LOS) measured in days. Secondary outcomes included: (1) postoperative length of stay (defined as days from surgery to discharge), (2) time to first bowel movement, (3) time to first flatus, and (4) 30-day readmission rate. 

Data for primary and secondary outcomes were retrieved from the EHR, except for time to first flatus during Period one, which was recorded via patient questionnaire. All enrolled patients during that cycle completed this questionnaire. For patients with stomas in Period two, the time to first bowel movement was based on the first recorded stoma output.

Qualitative feedback was obtained from patients, nurses, residents, and surgeons throughout the study period to assess implementation feasibility and identify barriers to routine clinical integration. These qualitative insights were not formally recorded or analyzed but informed iterative adjustments to the protocol.

Estimated cost savings were calculated using publicly available estimates of average hospital day costs in Minnesota, as reported by the American Hospital Association, as well as the CPT code 98928 (OMT; 7-8 body regions involved) [24]. Direct per-patient costs were not calculated.

Analysis

Data were de-identified and compiled in XLSX format in Excel (Microsoft Corporation, Redmond, Washington). Analysis and data visualization were conducted in R version 4.4.2 (R Foundation for Statistical Computing, Vienna, Austria). The analysis included all participants. Data normality was assessed using both visual methods and the Shapiro-Wilk test [25]. All data were available, and thus no concessions or alterations were made for missing data during preparation or analysis. 

Baseline differences between control and intervention groups were assessed via Welch's t-test and chi-squared tests. 

Length of stay, postoperative length of stay, days to first bowel movement, and days to first flatus were non-normal. Therefore, we used a Mann-Whitney U test, including a Hodges-Lehmann (HL) to quantify median differences and rank-biserial correlations to describe effect size (r) [26]. Differences in 30-day readmission rates were analyzed using Fisher’s exact test.

Subgroup analysis based on surgery type (open vs laparoscopic/robotic) was also conducted using a Mann-Whitney U test, including a Hodges-Lehmann (HL) to quantify median differences and rank-biserial correlations to describe effect size (r). 

A sensitivity analysis was conducted to assess the reliability of the primary findings [27]. Primary comparisons were repeated after applying 90% winsorization (5th and 95th percentiles) to mitigate the influence of outliers [28]. Winsorization removes extreme data by setting values above or below pre-specified percentiles to the value at that percentile. In this case, data below the 5th percentile was adjusted to the 5th percentile value, and data above the 95th percentile was adjusted to the 95th percentile value. This provides a symmetrical approach to outlier removal that avoids completely ignoring extreme data points. Additionally, Welch’s t-tests were conducted to examine whether results differed meaningfully under the assumption of normality.

Barriers to implementation

The main barrier to implementation was the availability of providers who could perform OMT, which limited the project to a single study site. No other significant barriers to implementation were identified before, during, or after the project.

## Results

**Figure 2:**
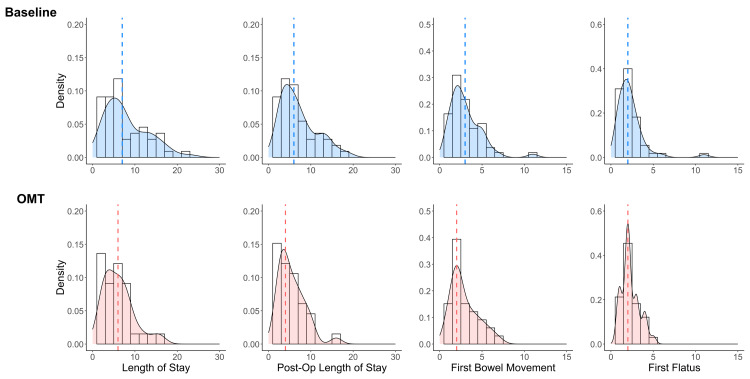
Distribution of outcomes in baseline and OMT groups Density plots comparing clinical outcomes in days between baseline (top row, blue) and OMT intervention (bottom row, red) groups. The OMT group demonstrates non-significant leftward shifts in length of stay, postoperative length of stay, and time to first bowel movement. Dashed lines indicate median values. All distributions are right-skewed.

The analysis involved 88 patients, including 20 prospective patients enrolled during Cycle 1, 13 during Cycle 2, and 55 retrospective comparison patients. Groups were comparable at baseline, with no significant difference in age, sex, body mass index (BMI), or surgery type (Table 2). All enrolled patients completed the treatment protocol and were included in the analysis. Normality tests revealed non-normal, right-skewed distributions for all outcome variables (Figure 2).

**Table 2:** Patient demographics Patient demographics in baseline and OMT groups. Continuous variables are reported as *mean *(SD). Dichotomous variables are reported as *n* (%). Baseline differences were assessed using a t-test for continuous and chi-squared test for dichotomous variables. SD: standard deviation.

	Baseline, n = 55	OMT, n = 33	p-value
Age in years, mean (SD)	64.3 (13.8)	64.4 (14.4)	0.989
Sex, number (%)			0.956
Male	25 (45%)	14 (42%)	
Female	30 (55%)	19 (58%)	
Body mass index, mean (SD)	29.4 (7.37)	30.7 (8.5)	0.469
Surgery, number (%)			0.525
Open	43 (78%)	23 (70%)	
Laparoscopic/Robotic	12 (22%)	10 (30%)	

Primary outcome

**Figure 3:**
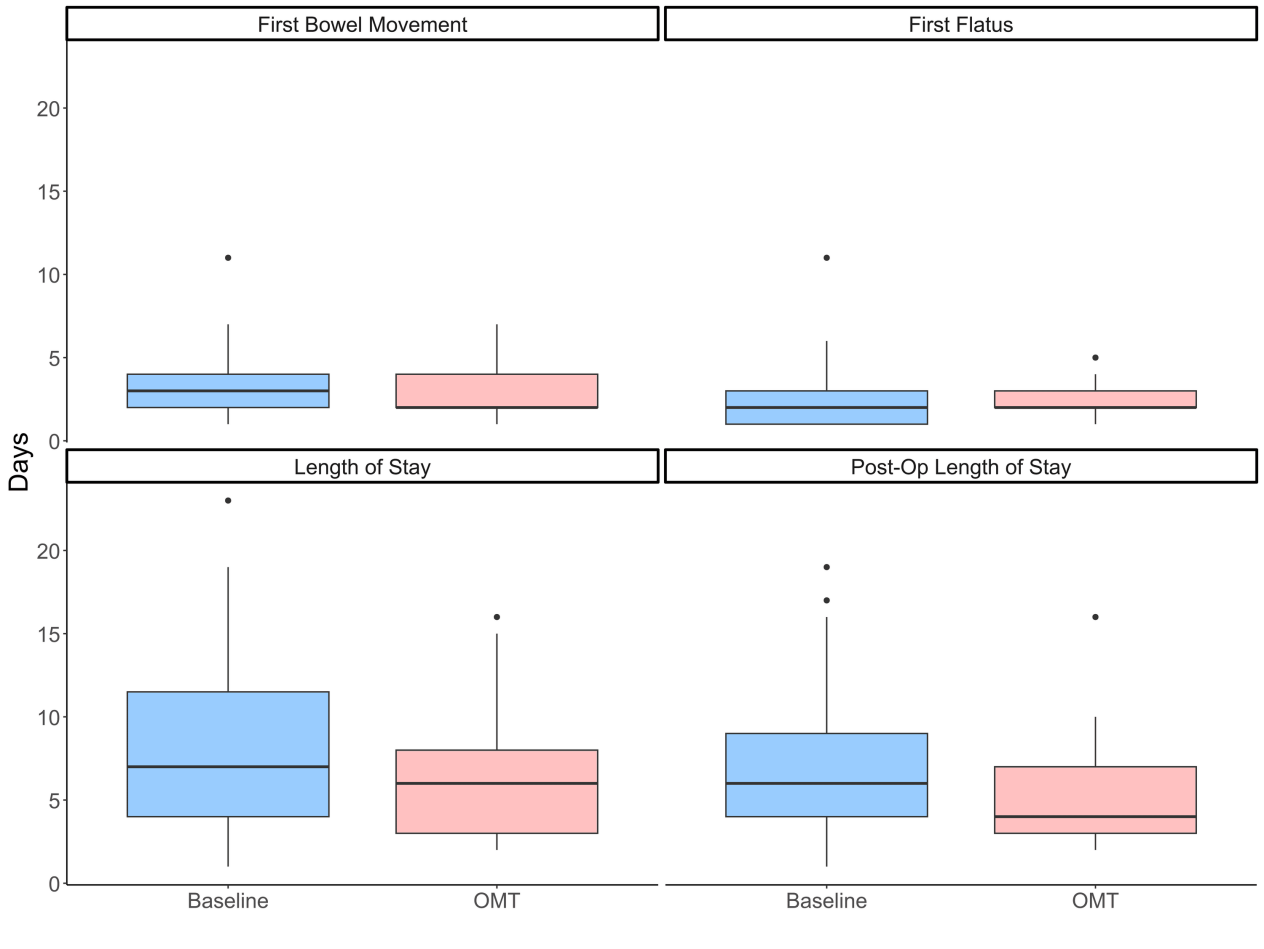
Comparison of outcomes in baseline and OMT groups The OMT group (red) shows a non-significant reduction in length of stay and postoperative length of stay compared to baseline (blue). Boxes show IQR with the central line at the median. Whiskers represent the furthest data point within 1.5 IQR. Data points falling outside 1.5 IQR are plotted individually.

The median hospital LOS in the baseline group was 7.0 days [interquartile range (IQR)=7.5] compared with 6.0 (IQR=5.0) in the OMT group (Figure 3). The mean LOS was 8.00 [standard deviation (SD)=5.03] days in the baseline group and 6.15 (SD=3.57) days in the OMT group (Figure 3). The OMT group showed a non-significant reduction in median length of stay compared to the baseline group. The effect size, as measured by the rank-biserial correlation, was small to moderate and favored the OMT group (Table 3).

**Table 3:** Results of primary and secondary outcome analysis Mann-Whitney U tests results are shown for each variable including a Hodges-Lehmann estimator and corresponding confidence interval. Raw median difference is also reported. Additionally, effect sizes (*r*) have been calculated with rank-biserial correlation and are displayed with a corresponding confidence interval.

	Mann-Whitney U Test				Rank-Biserial Correlation	
Outcome	Median Difference	Hodges-Lehmann Estimator	95% CI [Lower, Upper]	p-value	r	95% CI [Lower, Upper]
Length of Stay (Primary)	-1.0	-1.00	[-3.00, 0.00]	0.128	-0.19	[-0.44, 0.03]
Time to First Bowel Movement	-1.0	0.00	[-1.00, 0.00]	0.769	-0.04	[-0.28, 0.21]
Time to First Flatus	0.0	0.00	[-0.00, 0.00]	0.413	-0.10	[-0.15, 0.37]
Postoperative Length of Stay	-2.0	-1.00	[-3.00, 0.00]	0.100	-0.21	[-0.43, 0.04]

Secondary outcomes

The median postoperative LOS was 6.0 (IQR=5.0) in the baseline group and 4.0 (IQR=4.0) in the OMT group. The mean postoperative length of stay was 7.11 (SD=4.28) and 5.52 (SD=3.11) in the baseline and OMT groups, respectively.

The median time to first bowel movement was 3.0 days (IQR=2.0) in the baseline group versus 2.0 days (IQR=2.0) in the OMT group. Corresponding mean values were 3.56 days (SD=2.02) and 2.91 days (SD=1.61), respectively.

The median time to first flatus was 2.0 days (IQR = 2.0) in that baseline group and 2.0 days (IQR=1.0) in the OMT group. The mean time to first flatus was 2.27 days (SD=1.62) in the baseline group and 2.30 days (SD=1.04) in the OMT group (Table 2).

Non-significant reductions in postoperative LOS, time to first flatus, and time to first bowel movement were observed in the OMT group. Postoperative LOS had a small-to-moderate effect size favoring OMT, while time to first flatus and time to first bowel movement did not show substantial effect sizes (Table 2).

At 30 days, 9 of 55 patients (16%) in the baseline group and 2 of 33 patients (6%) in the OMT group were readmitted. The difference in readmissions was non-significant (p=0.198).

Subgroup analysis 

Subgroup analysis conducted by surgery type revealed significant differences in median length of stay and median postoperative length of stay in patients undergoing open surgical procedures (Table 4). Both outcomes also showed statistically significant, moderate-to-large effect sizes favoring the OMT group. No significant difference was found for time to first bowel movement or time to first flatus in the open subgroup. No significant differences were found for any outcome in the laparoscopic/robotic subgroup. 

**Table 4:** Subgroup analysis based on surgery type Mann-Whitney U tests results are shown for each variable including a Hodges-Lehmann estimator and corresponding confidence interval. Raw median difference is also reported. Additionally, effect sizes (r) have been calculated with rank-biserial correlation and are displayed with a corresponding confidence interval. Asterisk (*) indicates significance.

	Mann-Whitney U Test					Rank-Biserial Correlation	
Subgroup	Outcome	Median Difference	Hodges-Lehmann Estimator	95% CI [Lower, Upper]	P-value	r	95% CI [Lower, Upper]
Open (control = 43, OMT = 23)	Length of Stay (Primary)	-1	-2.99	[-5.00, -1.00]	0.011*	-0.38	[-0.60, -0.10]
	Time to First Bowel Movement	-1	0.00	[-1.00, 1.00]	0.578	-0.83	[-0.36, 0.21]
	Time to First Flatus	0	0.00	[0.00, 0.99]	0.455	0.11	[-0.18, 0.38]
	Postoperative Length of Stay	-2	-2.00	[-3.99, 0.00]	0.028*	-0.33	[-0.56, -0.04]
Laparoscopic/Robotic (control = 12, OMT = 10)	Length of Stay (Primary)	3	2.00	[-0.00, 0.00]	0.102	0.42	[-0.05, 0.73]
	Time to First Bowel Movement	0	0.00	[-1.00, 1.00]	0.552	0.15	[-0.33, 0.57]
	Time to First Flatus	0	0.00	[-0.00, 1.00]	0.451	0.18	[-.30, 0.59]
	Postoperative Length of Stay	1	1.00	[-1.00, 3.00]	0.269	0.28	[-0.20, 0.66]

Sensitivity analysis

Sensitivity analyses using 90% winsorization supported the findings of the primary analysis. Parametric testing revealed no changes in statistical significance in time to first bowel movement or time to first flatus. Total length of stay and postoperative length of stay were found to be significant when applying parametric methods; however, these results should be interpreted with caution and treated as exploratory only (Table 5).

**Table 5:** Results of primary and secondary outcome sensitivity analysis Mann-Whitney U tests results are shown for each variable including a Hodges-Lehmann estimator and corresponding confidence interval. Raw median difference is also reported. Additionally, exploratory parametric analysis was conducted using Welch's t-test. Asterisk (*) indicate statistical significance.

	Mann-Whitney U Test (Winsorized)				Welch’s t-Test
Outcome	Median Difference	Hodges-Lehmann Estimator	95% CI [Lower, Upper]	p-value	p-value
Length of Stay (Primary)	-1.0	-1.00	[-3.00, 0.00]	0.109	0.048*
Time to First Bowel Movement	-1.0	0.00	[-1.00, 1.00]	0.780	0.700
Time to First Flatus	0.0	0.00	[-0.00, 0.00]	0.424	0.915
Postoperative Length of Stay	-1.0	-1.00	[-3.00, 0.00]	0.080	0.047*

Stakeholder feedback, pitfalls, and potential cost savings

Based on stakeholder feedback, the protocol was modified for Cycle 2. No significant difference in length of stay was found between Cycle 1 and Cycle 2, despite the change in treatment protocol [median difference=0 days, Hodges-Lehmann estimator: 0, 95% CI (−2.99, 2.0), p=0.99].

The additional cost of osteopathic treatment was an estimated average of $408 per patient, assuming the median length of stay for the OMT group (6.0 days). A potential cost saving of approximately $2,745 per patient was projected based on a one-day median LOS reduction, yielding a net cost benefit of $2,337.

No significant adverse events were reported during implementation of the osteopathic treatment protocol.

## Discussion

This quality improvement project successfully achieved its primary aim of reducing both total length of stay and postoperative length of stay in patients at risk of POI, though observed reductions did not reach statistical significance in the primary outcome. This intervention did not show increased risk of complications and likely introduced cost savings. Clinical staff including nurses and surgeons reported no disruptions to workflow or perceived additional burden resulting from OMT implementation. No logistical challenges or safety concerns were identified and the treatment protocol had excellent adherence during the intervention periods, with no patients missing treatments.

Despite a lack of statistical significance in the primary and secondary outcomes, the calculated median differences, Hodges-Lehmann statistics, and r values agree in direction and magnitude suggesting a clinically relevant 14% and 33% reduction in LOS and postoperative LOS respectively. Additionally, parametric sensitivity analysis showed significant effects in both outcomes, supporting the plausibility of the effect though this analysis is purely exploratory. Furthermore, significance would appear to be fragile and reliant on underlying statistical assumptions, indicating that the decision to conduct non-parametric analysis was influential and that this result should be interpreted with caution.

Notably, subgroup analysis did reveal statistically significant benefit in patients undergoing open surgical procedures. Both LOS and postoperative LOS showed significant reductions and had moderate-large effect sizes favoring the OMT group. This would indicate that, at least for this subset of patients, the protocol implemented was associated with both a clinically relevant and statistically significant 16% reduction in overall LOS and 28% reduction in postoperative LOS . Additionally, while readmission rates were not significantly different between OMT and control groups, given low sample sizes, there exists a possibility of type II error. Thus the large difference in readmissions seen here, again favoring OMT, may represent a clinically significant phenomenon that may reach statistical significance in a larger study. 

These results align with a growing body of literature suggesting that OMT may be a safe and cost-effective adjunct in postoperative care. For instance, in a 2008 retrospective study of 331 cases of POI, patients who received OMT treatment had a significantly shorter hospital duration (11.8 days compared to the non-treatment group average of 14.6 days) [8]. Another retrospective study in 2013 reviewed 55 abdominal surgery cases, 17 of which received OMT treatment, and found statistically significant reductions in time to first flatus and length of stay [5]. A more recent 2024 meta-analysis reported a mean reduction in hospital stay of 2.37 days across studies utilizing OMT, although the pooled estimate did not reach statistical significance [29]. Combined with the present findings, this body of evidence supports further investigation into the utility of OMT as a supportive therapy in postoperative recovery, though it does not provide definitive proof of efficacy.

Importantly, this intervention was implemented without substantial added complexity or adverse events. Informal feedback from staff indicated high adherence and positive patient response, and the financial analysis presented here suggests the potential for meaningful cost savings. Additionally, prior survey-based data has shown that OMT provides consistent improvement in a number of patient centered metrics [30]. These effects, while more difficult to quantify, further support the inclusion of OMT in multimodal recovery protocols and are reflective of the informal feedback received from participants throughout the project period.

Several limitations reduce the generalizability of this project, including those attributable to the design characteristics of QI studies. For instance, no attempt at randomization or blinding took place and no analysis plan, including power analysis, was established prior to data collection. Additionally, this was a low sample size, single center study with the intervention performed by residents. This limits external validity and generalizability. What's more, because patients were enrolled via convenience sampling, selection bias is difficult to account for when interpreting results. While these limitations do not undermine the potential relevance of the observed reduction in hospital stay, they do constrain the interpretability of inferential statistics. As such, the results should not be interpreted as evidence for or against a causal relationship. Instead, these findings should be considered exploratory and warrant further evaluation in rigorously designed prospective studies.

Despite these limitations, the results of this QI project suggest that this protocol for reducing POI may improve patient care and save system resources. The relatively brief treatment time and low cost of administration make this an attractive choice for reducing postoperative LOS, if trained osteopathic providers are available. Osteopathic manipulative treatment in general is a cost effective and low-risk intervention. Thus, given the lack of significant preventative measures for POI, the protocol presented here warrants further investigation.

Future studies based on this work could explore a number of questions. It is not clear which, if any, OMT techniques directly influence the pathophysiological mechanisms of POI. While the protocol presented here is based on the current understanding and theory of OMT, there exists a knowledge gap in technique-specific mechanisms of action. A better understanding of this could lead to more targeted technique selection and refinement of this protocol. Additionally, there are likely patient-level factors that were excluded from our analysis. This project did not attempt to control for surgical technique, procedure, patient comorbidities, or any other factor that might influence outcomes. Ideally, given the positive but non-significant findings presented here, a follow-up study might include a larger sample size, proper randomization, and more control over confounding variables. Ultimately, for a protocol such as this to be implemented on a wide scale, more robust clinical data is needed.

## Conclusions

This QI project suggests that the proposed OMT protocol is associated with a reduction in LOS and postoperative LOS in patients undergoing open abdominal surgery who are at risk for postop ileus as evidenced by subgroup analysis. However, primary and secondary outcomes did not reach statistical significance and due to study limitations, a causal relationship cannot be established. More research is required to determine if the trends identified here represent meaningful clinical effects.

## Figures and Tables

**Figure 4:**
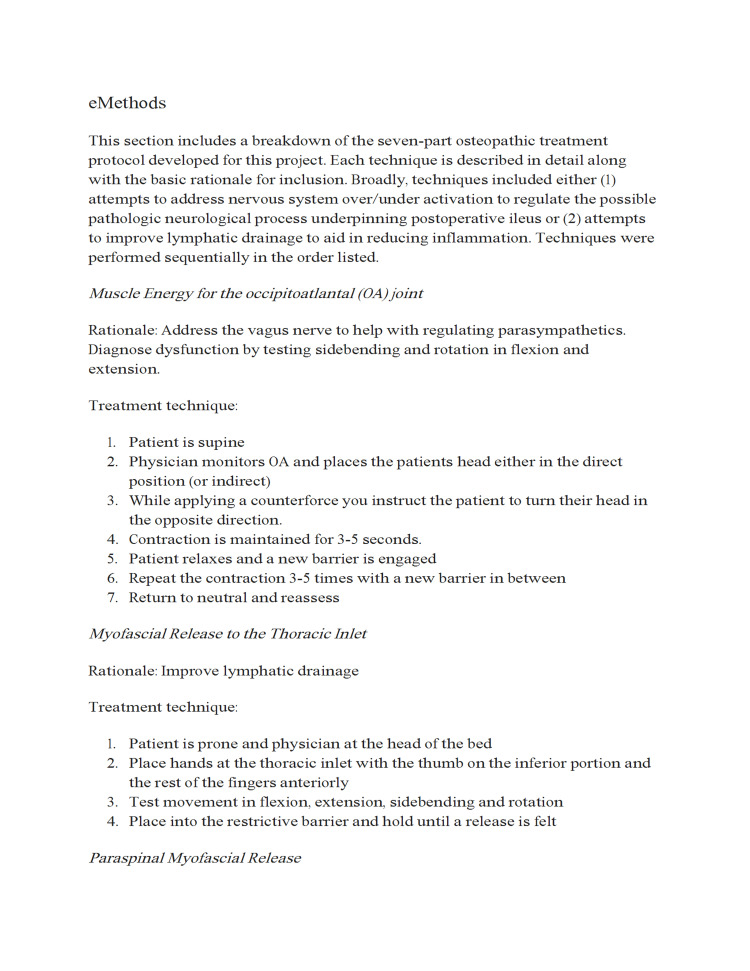
eMethods - part 1

**Figure 5:**
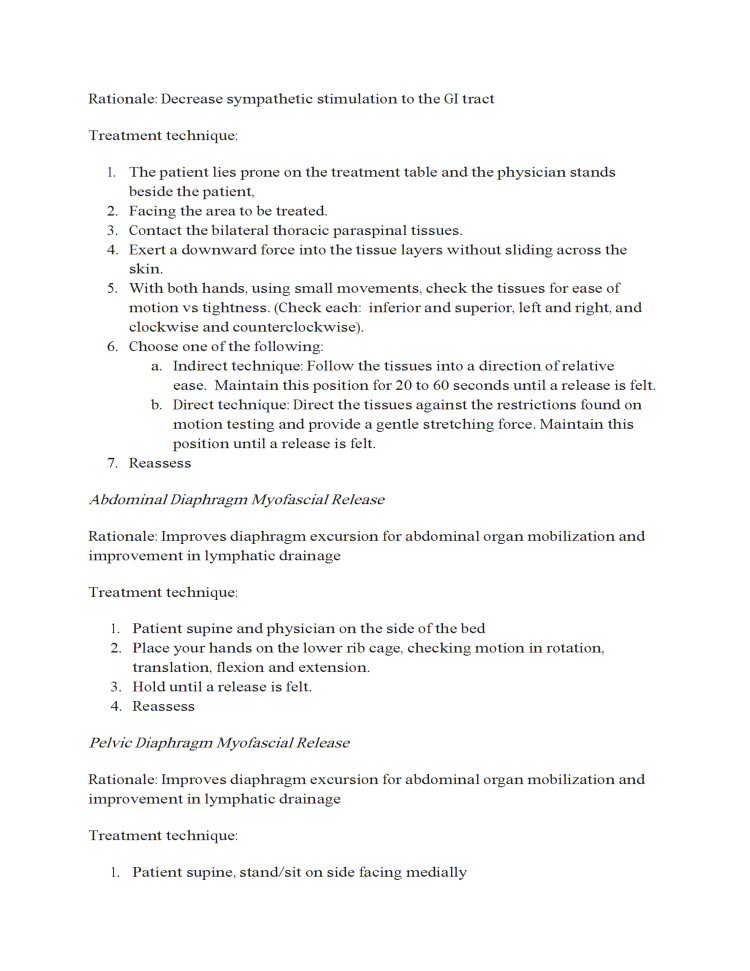
eMethods - part 2

**Figure 6:**
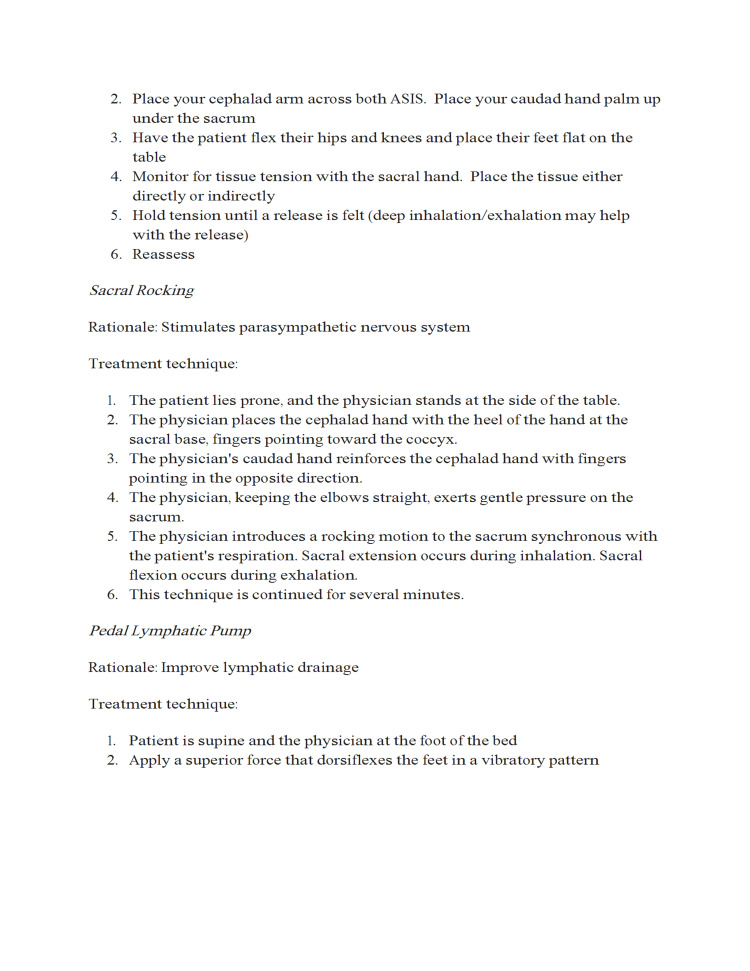
eMethods - part 3

## Appendices

Appendix 1 

Appendix 2 

Appendix 3 
